# Treatment of Atypical Hemolytic-Uremic Syndrome in the Era of Eculizumab

**DOI:** 10.7759/cureus.1111

**Published:** 2017-03-23

**Authors:** Rawaa Ebrahem, Salam Kadhem, Quoc Truong

**Affiliations:** 1 Internal Medicine, University of Kansas School of Medicine-Wichita; 2 Cancer Center of Kansas

**Keywords:** microangiopathic hemolytic anemia, thrombocytopenia, acute kidney injury, eculizumab, duration of treatment

## Abstract

Hemolytic-uremic syndrome (HUS) is the triad of microangiopathic hemolytic anemia (MAHA), thrombocytopenia, and acute kidney injury (AKI); the main cause of multi-organ failure is related to thrombotic microangiopathy (TMA). Atypical HUS (aHUS) is a disease of uncontrolled complement activation associated with a high mortality rate and most cases progress to end-stage renal disease. About 50% of patients with this syndrome carry mutations in genes that encode complement proteins. Also, aHUS constitutes an over-activation of the complement pathway which is either inherited, acquired, or both. This results in TMA. Plasma infusions or exchange should be performed daily until the platelet count, lactate dehydrogenase (LDH), and hemoglobin levels are substantially improved, or until an alternate treatment strategy has been decided upon. Eculizumab (a terminal complement inhibitor approved in 2011 for treating aHUS) treatment should begin immediately when the diagnosis is confirmed. There is limited evidence on the duration of the treatment despite significant clinical interest in investigating this aspect. Therefore, it is crucial to conduct further analysis on the possible dose and time adjustments.

## Introduction

Hemolytic-uremic syndrome (HUS) is a triad of microangiopathic hemolytic anemia (MAHA), thrombocytopenia, and acute kidney injury (AKI) [1]; the related multi-organ failure is secondary to thrombotic microangiopathy (TMA).

Atypical HUS (aHUS), which is caused by complement dysregulation and over-activation of the alternative pathway, eventually causes either a loss of function mutation in a regulatory gene (complement factor H (CFH), complement factor I (CFI), or cluster of differentiation 6 (CD6)) or a gain of function mutation in an effector gene (complement factor B (CFB) or C3); there is normal von Willebrand factor-cleaving protease (ADAMTS13) activity which is inconsistent with other differential diagnoses, such as thrombotic thrombocytopenic purpura (TTP) [2].

The atypical version of the syndrome may be triggered by infection, pregnancy, or autoimmune disease which results in the activation of the complement system and the formation of a membrane attack complex (MAC) [3]. Eculizumab, a terminal complement inhibitor, is a humanized monoclonal antibody that binds with high affinity to the C5 protein and blocks the generation of C5a and C5b. It has been proven as an effective therapy for aHUS [4].

We report the case of a 31-year-old female with aHUS who was prescribed eculizumab for a total duration of one year; then, therapy was stopped without any relapse or complications.

## Case presentation

A 31-year-old African American female with past medical history significant for type I diabetes, uncontrolled hypertension, chronic kidney disease, venous thrombosis, and cerebral palsy presented with a severe headache, confusion, cough, dyspnea, bilateral lower extremity edema, and pain. One month prior, she had had a history of pneumonia refractory to antibiotics treatment. Upon her admission, labs were significant for severe anemia (Hb of 6.4 g/dl), thrombocytopenia (platelets between 100,000 and 120,000), elevated lactate dehydrogenase (LDH) ( > 5000 U/L), creatinine of 3.4 mg/dl, and five percent schistocyte on a peripheral blood smear. The combination of MAHA, AKI, and thrombocytopenia in addition to a headache and confusion at the time of the presentation confirmed the diagnosis of aHUS/TTP.

In consultation with a hematologist, the patient was placed on daily plasma exchange. She initially improved over the first 48 hours, demonstrated by decreased LDH and increased platelets. This was followed by gradual deterioration; however, her ADAMTS13 was normal. She was then started on eculizumab. A significant improvement was achieved by the second dose.

**Figure 1:**
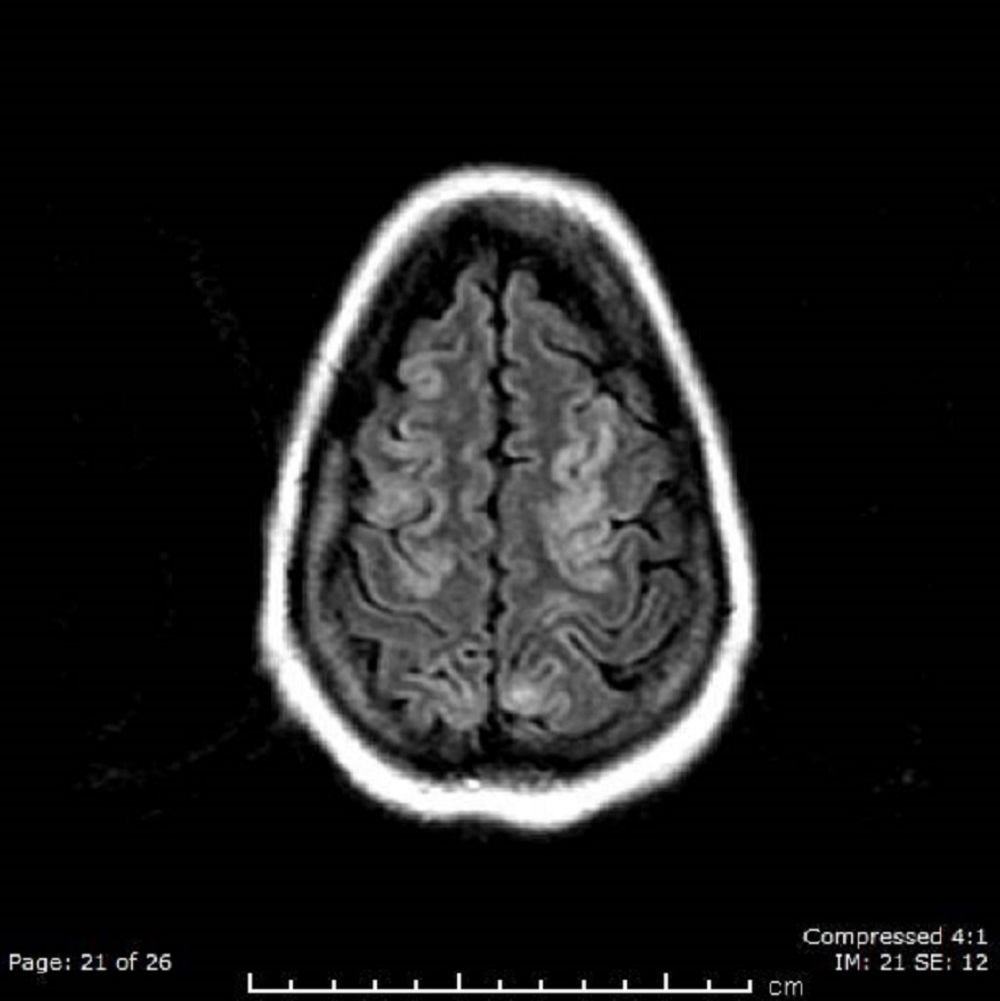
Brain MRI Shows Abnormal Signal at the Parieto-occipital Region

Her labs revealed that CFH, properdin factor B (Bf), CFI, and CFB were negative and that ADAMTS13 was > 100; in addition, C3 and C4 levels were normal, antiphospholipid and lupus screenings were negative. Genetic tests for complement factor H-related protein 5 (CFHR5), membrane cofactor protein (CD46 or MCP) and encoding for thrombomodulin (THBD) were not significant and revealed normal alleles; however, she was found to have complement factor H-related protein 3 - complement factor H-related protein 1 (CFHR3-CFHR1) gene deletion, which is three times more common in aHUS patients than in the general population. A brain magnetic resonance imaging (MRI) scan revealed there were gyriform areas of abnormal signal bilaterally in the parieto-occipital, fronto-parietal, and occipital regions (Figure 1).

Differential diagnoses would include vasculitis and/or encephalitis. These areas do show mild increased signal on diffusion-weighted images; subacute ischemic changes could also have this appearance. After being started on aspirin and eculizumab, the patient improved dramatically by the second dose. This medication regimen was continued for a total of one year before being stopped. The patient has not shown any evidence of relapse since that time.

## Discussion

aHUS constitutes an over-activation of the complement pathway which is either inherited, acquired, or both; it results in TMA, which would, in turn, cause multi-organ failure [5]. Complement-mediated HUS, which stems from the activation of the alternative pathway, includes CFH, CFI, and CFB, which check C3 and C4 in addition to CD46 and THBD [6]. In an acute presentation of aHUS, plasma exchange or infusion can be used to maintain platelets and low LDH; however, the underlying microangiopathic thrombosis and complement dysregulation is likely to persist [7]. The deletion of CFHR1 and CFHR3 is strongly associated with aHUS. CFHR1 binds to C3b and C5 and regulates the C5 convertase and terminal complement pathway; in case of the deletion of these two regulatory factors, an over-activation of the pathway and endothelial injury will result [8]. Eculizumab, a terminal complement inhibitor that binds to C5 with high affinity, blocks the generation of proinflammatory C5a and C5b-9. Approved in 2011 in the United States, it is the drug of choice for the treatment of aHUS; immediate treatment with eculizumab is strongly recommended when the diagnosis of aHUS is confirmed. Three recent meta-analyses revealed that eculizumab is clinically effective in patients with aHUS  [9-10]. It reduces TMA events, as reflected by normal platelets, and improves the quality of life by decreasing the need for dialysis. However, clear guidelines or studies to determine the duration of treatment are still lacking. In our patient, eculizumab was stopped after one year and no further TMA events were reported. Based on this, we may conclude that in certain scenarios, especially if the triggering factors are known, eculizumab therapy may be considered as a temporary treatment until resolution of symptoms is achieved.

## Conclusions

aHUS is a rare disease; however, as it is associated with high mortality rates and poor outcomes, administration of eculizumab is crucial. Due to the heterogeneity of aHUS and the cost of eculizumab, which places a significant burden on health care budgets, further studies are essential to decide the duration of the treatment. There is limited evidence on when to stop treatment despite the significant clinical interest in investigating this possibility. Therefore, it is crucial to conduct further analyses for possible dose adjustments and the option of treatment cessation when clinicians consider it appropriate.
